# Toward targeted dementia prevention: Population attributable fractions and risk profiles in Germany

**DOI:** 10.1002/dad2.70225

**Published:** 2025-11-26

**Authors:** Iris Blotenberg, Jochen René Thyrian

**Affiliations:** ^1^ German Center for Neurodegenerative Diseases (DZNE) Greifswald Germany; ^2^ Brigham and Women's Hospital Harvard Medical School Boston Massachusetts USA; ^3^ Institute for Community Medicine Greifswald Germany

**Keywords:** Alzheimer's disease, cognition, cognitive decline, lifestyle, modifiable risk factors, prevention, risk groups

## Abstract

**INTRODUCTION:**

Effective dementia prevention requires understanding the distribution of modifiable risk factors and identifying high‐risk subgroups. We estimated the prevention potential in Germany and identified risk profiles to inform precision public health.

**METHODS:**

We analyzed nationally representative data from the 2023 German Aging Survey (*n* = 4992). Population attributable fractions and potential impact fractions were computed for established modifiable risk factors. Relative risks were taken from meta‐analyses. Latent class analysis identified risk profiles.

**RESULTS:**

An estimated 36% of dementia cases in Germany are attributable to modifiable risk factors. Reducing their prevalence by 15%–30% could prevent 170,000–330,000 cases by 2050. We identified four risk profiles—metabolic, sensory impairment, alcohol, and lower‐risk—each associated with demographic and regional characteristics.

**DISCUSSION:**

Our findings highlight considerable national prevention potential and reveal population subgroups with shared risk patterns. These profiles provide a foundation for designing targeted, equitable, and efficient dementia prevention strategies.

**Highlights:**

- 36% of dementia cases in Germany are linked to modifiable risk factors.
- A 15% reduction in risk factor prevalence could prevent 170,000 cases by 2050.
- Key contributors: depression, hearing loss, low education, and obesity.
- Data‐driven risk profiles identified (e.g., metabolic, sensory, low‐risk).
- Risk profiles strongly associated with sociodemographic characteristics.

## 1 INTRODUCTION

Our understanding of modifiable dementia risk factors is rapidly evolving. The Lancet Commission's updated report identified 14 modifiable risk factors across the lifespan, estimating that up to 45% of dementia cases worldwide may be linked to these factors.^1^ The updated Lancet Commission report also presents revised estimates of relative risks, with higher values for depression and diabetes and lower ones for hearing loss, obesity, hypertension, and smoking than in previous reports.^2^, ^3^

**RESEARCH‐IN‐CONTEXT**

1. **Systematic review**: We searched PubMed for studies on dementia prevention in Germany. Prior estimates relied on older data and outdated relative risk values. To date, no studies have systematically identified population‐level dementia risk profiles in Germany using recent national data.
2. **Interpretation**: Over one‐third of dementia cases in Germany are attributable to modifiable risk factors. These are unevenly distributed across the population, forming distinct risk profiles—such as metabolic, sensory, and alcohol‐related clusters—closely tied to sociodemographic characteristics (e.g., age, sex, education, region).
3. **Future directions**: Research should assess whether similar subgroups exist in other populations. In Germany, these profiles provide a data‐driven basis for precision prevention. Public health interventions should be tailored to profile‐specific needs and barriers, and their impact evaluated in targeted trials.

In Germany, an estimated 1.8 million people are currently living with dementia.^4^, ^5^ Without effective preventive measures, this number could rise to 2.7 million by 2050.^5^ In light of this, an up‐to‐date assessment of the national prevention potential is urgently needed. The release of new data from the 2023 wave of the German Aging Survey (DEAS)—a nationally representative cohort—now enables such an assessment based on current prevalence rates and updated risk estimates.^6^ The first aim of this study is to quantify the proportion of dementia cases in Germany attributable to modifiable risk factors and to estimate the potential impact of population‐level risk reduction strategies.

While multidomain interventions, such as the FINGER trial and its international adaptations,^7^, ^8^, ^9^, ^10^ have shown promise, such individual‐level measures tend to have limited “population impact” due to narrow reach and the neglect of structural and environmental causes of disease.^11^, ^12^ Although the assessment of the prevention potential at the national level is crucial, a universal or large‐scale rollout of preventive strategies would be highly resource‐intensive and risk misallocating efforts. Given the heterogeneity of dementia risk across the population,^13^ a more effective approach may lie in identifying and targeting empirically defined subgroups that share distinct constellations of modifiable risk factors.

There is a lack of studies using nationally representative data to systematically identify such dementia risk profiles in the population. The second aim of our study is therefore to derive data‐driven risk subgroups using latent class analysis (LCA) and to examine their sociodemographic correlates. This approach can inform more precise, feasible, and equitable public health strategies and may provide a model for risk stratification in other countries.

## 2 METHODS

### 2.1 Risk factors and sociodemographic predictors

This study utilized data from the most recent wave (2023) of the DEAS, published in March 2025 (*n* = 4992).^6^ The DEAS is a nationwide representative cross‐sectional and longitudinal survey of the German population aged 40 years and older. Participants were interviewed either in person or by telephone using structured questionnaires and completed a written self‐report. Of the 14 modifiable risk factors identified by the *Lancet Commission*, 12 were assessed; data on traumatic brain injury and air pollution were not available. Air pollution exposure could only be roughly approximated using district type. For the risk factors hearing loss and depression, additional data sources were used. Specifically, an additional study on lifetime prevalence was included for depression,^14^, and an epidemiological study based on pure‐tone audiometric assessments was used for hearing loss.^15^ Table S1 in the Supplement provides an overview of the included risk factors. Sociodemographic characteristics included age, sex, district type, region (East/West), and cohabitation status.

### 2.2 Statistical analysis

#### 2.2.1 Prevention potential

Prevention potential was estimated following the Lancet Commission's approach,^1^, ^3^ using adjusted population attributable fractions (PAFs) via a modified Levin's formula.^16^ Relative risks were drawn from the most recent Lancet report;^1^ prevalence estimates from DEAS 2023.^6^ Post‐stratified weights based on the 2023 Microcensus were applied to ensure representativeness. Weighting factors adjusted for risk factor intercorrelations (e.g., metabolic syndrome components). A principal component analysis (PCA) of the tetrachoric correlation matrix was used to derive the weights, defined as 1 – communality for each risk factor. Potential impact fractions (PIFs) were also calculated to estimate case reductions under hypothetical risk factor reductions of 15% and 30%.

#### 2.2.2 Identification and characterization of risk profiles

Profiles of risk factors were identified using LCA. This statistical method, based on a structural equation modeling framework, allows for the identification of homogeneous subgroups within a heterogeneous sample. We applied full information maximum likelihood (FIML) to handle missing data in the risk factor variables. We tested models with one to five classes. A total of 11 dementia‐related risk factors—including all health and lifestyle factors—were used to define the LCA. The risk factor low education was included as a covariate, along with age, sex, living situation, district type, and region (eastern or western Germany). A multinomial logistic regression was performed to examine the associations between these covariates and the latent risk profiles, with the aim of identifying public health‐relevant subgroups. Model selection was based on the Akaike Information Criterion (AIC), Bayesian Information Criterion (BIC), sample‐size adjusted BIC (ssABIC), Vuong–Lo‐Mendell–Rubin likelihood ratio test (VLMRT), and the Lo‐Mendell–Rubin adjusted likelihood ratio test (LMRT), as well as interpretability. All analyses were conducted using Mplus Version 8.11.^17^

## 3 RESULTS

### 3.1 Prevention potential

Table 1 shows the prevalence values, relative risks, communalities, PAFs and PIFs for 12 of the 14 potentially modifiable risk factors for dementia identified by the Lancet Commission. Overall, the potential for dementia prevention in Germany was estimated at 36%. When including the risk factor air pollution, which could only be approximated by district type, the prevention potential increased to 39% (see Table S2). The most influential risk factors were depression, hearing loss, low educational attainment, obesity, and diabetes.

**TABLE 1:** Prevalences, relative risks, commonalities, PAF, and PIF (for a 15% or 30% prevalence reduction) for 12 potentially modifiable risk factors

Risk factor	Prevalence in the population in Germany, %	Relative risk (95% CI)	Communality, (%)	PAF, % (95% CI)	Adjusted PAF, % [95% CI]	15% Reduction of risk factor prevalence		30% Reduction of risk factor prevalence	
						PIF, % (95% CI)	Adjusted PIF, % (95% CI)	PIF, % (95% CI)	Adjusted PIF, % (95% CI)
Less education	15.4	1.6 (1.3‐2.0)	46.2	8.5 (4.4‐13.3)	3.3 (2.0‐4.5)	1.3 (0.7‐2.0)	0.6 (0.3‐0.9)	2.5 (1.3‐4.0)	1.1 (0.6‐1.7)
Hearing loss	23.3^a^ ‐ 40.6^b^	1.4 (1.0‐1.9)	56.7	14.0 (0.0‐26.8)	5.4 (0.0‐9.1)	2.1 (0.0‐4.0)	0.9 (0.0‐1.8)	4.2 (0.0‐8.0)	1.8 (0.0‐3.4)
High LDL cholesterol	23.9	1.3 (1.3‐1.4)	43.5	6.7 (6.7‐8.7)	2.6 (3.0‐3.0)	1.0 (1.0‐1.3)	0.5 (0.5‐0.6)	2.0 (2.0‐2.6)	0.9 (1.0‐1.1)
Depression	10.3^c^ – 15.9^d^	2.2 (1.7‐3.0)	59.2	16.0 (10.0‐24.1)	6.2 (4.5‐8.2)	2.4 (1.5‐3.6)	1.1 (0.7‐1.6)	4.8 (3.0‐7.2)	2.1 (1.4‐3.0)
Physical inactivity	20.7	1.2 (1.2‐1.3)	33.3	4.0 (4.0‐5.8)	1.5 (1.8‐2.0)	0.6 (0.6‐0.9)	0.3 (0.3‐0.4)	1.2 (1.2‐1.8)	0.5 (0.6‐1.0)
Smoking	22.3	1.3 (1.2‐1.4)	55.3	6.3 (4.3‐8.2)	2.4 (1.9‐2.8)	0.9 (0.6‐1.2)	0.4 (0.3‐0.5)	1.9 (1.3‐2.5)	0.8 (0.6‐1.0)
Diabetes	11.1	1.7 (1.6‐1.8)	51.3	7.2 (6.2‐8.2)	2.8 (2.8‐2.8)	1.1 (0.9‐1.2)	0.5 (0.5‐0.5)	2.2 (1.9‐2.4)	1.0 (0.9‐1.0)
Hypertension	34.5	1.2 (1.1‐1.4)	60.0	6.5 (3.3‐12.1)	2.5 (1.5‐4.1)	1.0 (0.5‐1.8)	0.4 (0.2‐0.8)	1.9 (1.0‐3.6)	0.9 (0.5‐1.5)
Obesity	26.7	1.3 (1.0‐1.7)	59.5	7.4 (0.0‐15.7)	2.9 (0.0‐5.4)	1.1 (0.0‐2.4)	0.5 (0.0‐1.0)	2.2 (0.0‐4.7)	1.0 (0.0‐2.0)
Alcohol consumption	29.2	1.2 (1.0‐1.5)	60.7	5.5 (0.0‐12.7)	2.1 (0.0‐4.3)	0.8 (0.0‐1.9)	0.4 (0.0‐0.8)	1.7 (0.0‐3.8)	0.7 (0.0‐1.6)
Social isolation	8.9	1.6 (1.3‐1.8)	31.8	5.1 (2.6‐6.6)	2.0 (1.2‐2.3)	0.8 (0.4‐1.0)	0.3 (0.2‐0.4)	1.5 (0.8‐2.0)	0.7 (0.4‐0.8)
Visual impairment	12.1	1.5 (1.4‐1.6)	62.0	5.7 (4.6‐6.8)	2.2 (2.1‐2.3)	0.9 (0.7‐1.0)	0.4 (0.3‐0.4)	1.7 (1.4‐2.0)	0.8 (0.7‐0.8)
Total					**35.7 (20.6‐50.8)**		**6.3 (3.4‐9.8)**		**12.2 (6.6‐18.7)**

*Notes*: Commonality represents the proportion of shared variance of the risk factor in question and the other risk factors. Percentages are based on exact values, minor deviations from the total are due to rounding.

Abbreviation: CI, confidence interval; LDL, low density lipoprotein; PAF, population attributable fractions; PIF, potential impact fractions.

^a^ Self‐reported hearing impairment from the German Aging Survey^6^ (*n* = 4992).

^b^ Prevalence from Döge (27).

^c^ Point prevalence from the German Aging Survey.^6^

^d^ Lifetime prevalence from the NAKO Health Study (28).

**FIGURE 1:**
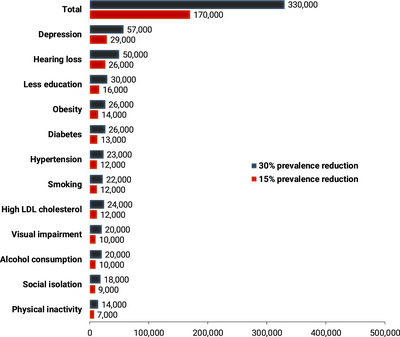
Number of dementia cases in 2050 that could theoretically be prevented if the population‐based prevalence of twelve modifiable risk factors were to be reduced by 15% or 30%. Basis for calculation: 2.7 million people with dementia in 2050

Figure 1 illustrates the projected number of dementia cases that could be prevented by 2050, if the population‐level prevalence of these risk factors were reduced by 15% or even 30%. A 15% reduction could lead to 170,000 fewer dementia cases by 2050 (from the projected 2.7 million cases), while a 30% reduction could potentially prevent up to 330,000 cases.

### 3.2 Risk profiles

**FIGURE 2:**
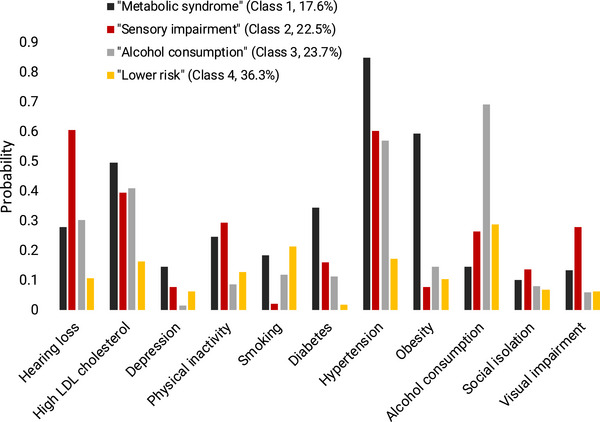
Probabilities of the presence of risk factors for each risk factor profile

Based on model fit indices, the four‐class model was selected as the best‐fitting solution (see Table S3 for the goodness‐of‐fit indices). The risk profiles of these four classes are presented in Figure 2. They were labeled according to the distribution of risk factors as follows: (1) The “metabolic syndrome” profile was characterized by the highest probabilities of hypertension, obesity, elevated LDL cholesterol, and diabetes. (2) The “sensory impairment” profile showed the highest probabilities for hearing loss and visual impairment. (3) The “alcohol consumption” profile had the highest probability of alcohol use. (4) The “lower‐risk” profile was characterized by probabilities below 0.3 for all risk factors.

#### 3.2.1 Characterization of risk profiles

The results of the multinomial logistic regression predicting risk profile membership are presented in Table 2. Individuals in the “metabolic syndrome” risk profile were older, had lower educational attainment and were more than twice as likely to live in eastern Germany than individuals in the “lower‐risk” profile. They were also more likely to live in smaller towns or rural districts. Individuals in the “sensory impairment” profile were also older, lived more frequently in eastern Germany, and were more likely to live in smaller towns than those in the “lower‐risk” profile. Finally, individuals in the “alcohol consumption” profile were older, more frequently male, and more likely to be cohabiting than those in the “lower‐risk” group. Table S4 shows further details on the sociodemographic characteristics of the four risk profiles.

**TABLE 2:** RRs and 95% CIs from the latent class analysis model with covariates using the lower risk group (Class 4) as the reference (*n* = 4989)

Variable	Class 1 "Metabolic syndrome“				Class 2 "Sensory impairment“				Class 3 "Alcohol consumption“			
		95% CI				95% CI				95% CI		
	RR	Lower	Upper	*p*‐Value	RR	Lower	Upper	*p*‐Value	RR	Lower	Upper	*p*‐Value
Age	1.082	1.041	1.125	**<0.001**	1.363	1.272	1.461	**<.001**	1.126	1.077	1.177	**<.001**
Sex												
Female	0.559	0.202	1.546	0.262	0.480	0.172	1.343	0.162	0.170	0.073	0.396	**<.001**
Education level												
Moderate education	*0.517*	*0.266*	*1.008*	***0.053***	1.807	0.588	5.551	0.302	0.872	0.299	2.544	0.803
High education	0.227	0.078	0.657	**0.006**	1.924	0.527	7.019	0.322	1.818	0.477	6.928	0.381
Living situation												
Cohabitating	0.887	0.516	1.526	0.665	1.093	0.593	2.015	0.775	3.042	1.739	5.322	**<.001**
Region												
Eastern Germany	2.313	1.594	3.356	**<0.001**	2.089	1.095	3.985	**0.025**	1.043	0.397	2.739	0.932
District type												
Independent large city (ref.)												
Urban district	1.507	1.044	2.175	**0.029**	1.842	1.003	3.383	**0.049**	1.483	0.683	3.216	0.319
Rural district with densification trends	1.241	0.826	1.865	0.299	1.524	0.714	3.252	0.276	1.062	0.432	2.613	0.895
Sparsely populated rural district	1.684	1.088	2.606	**0.019**	1.004	0.439	2.296	0.993	0.971	0.475	1.986	0.935

Abbreviations: CI, confidence interval; RR, relative risk.

## 4 DISCUSSION

In this nationally representative study, we show that there is a considerable prevention potential for dementia in Germany: an estimated 36% of dementia cases are attributable to 12 of the 14 modifiable risk factors identified by the Lancet Commission.^1^ This estimate is lower than the global figure of 45% reported by the Lancet Commission, which can be attributed to differences in the distribution of risk factors across countries and regions. It is consistent with findings from other country‐ or region‐specific analyses (e.g., ^18^, ^19^) and underscores the relevance of tailoring prevention strategies to specific population contexts. Notably, if a 15% reduction in risk factor prevalence were achieved in Germany, approximately 170,000 dementia cases could theoretically be prevented or delayed by 2050. A 30% reduction could yield more than 330,000 prevented cases. Moreover, such measures may also have broader effects on other conditions that share similar risk factors, such as heart disease, stroke, or cancer.^20^

Beyond the overall prevention potential, we identified four distinct population‐based risk profiles using LCA. These profiles—metabolic, sensory impairment, alcohol consumption, and lower‐risk—represent empirically derived constellations of modifiable dementia risk factors. To our knowledge, this is one of the first studies to identify such subgroups using nationally representative data. Importantly, the risk profiles were associated with sociodemographic characteristics such as age, education, and region, pointing toward structural differences in dementia risk exposure across the population. These findings lay a foundation for developing more targeted, subgroup‐specific prevention strategies that go beyond a universal, one‐size‐fits‐all model.

Several modifiable risk factors emerged as particularly influential: depression, hearing loss, low educational attainment, obesity, and diabetes. Many of these factors are influenced not only by individual behavior, but also by structural determinants of health. Effective dementia prevention in Germany will therefore require a combination of individual‐level behavioral interventions and broader system‐level strategies, such as improved access to mental health care, increased availability of hearing aids, and policies that reduce educational inequality. Education deserves special attention—not only as an independent protective factor, but also as a determinant of many other risk factors (e.g., see Puka et al^21^). In Germany, educational attainment is strongly associated with socioeconomic disparities.^22^ Enhancing educational equity may therefore serve as an important lever for dementia prevention.

Our findings indicate structurally rooted regional differences in dementia risk. For instance, individuals living in rural areas and eastern Germany were more likely to belong to high‐risk profiles, particularly the metabolic or sensory impairment risk profiles. These results are also consistent with the regional distribution of dementia cases in Germany, where—even after age standardization—a particularly high prevalence is found in the eastern federal states,^23^ which tend to be structurally disadvantaged.^24^ This knowledge of diverse dementia risk profiles and their sociodemographic patterns can inform more targeted and effective prevention strategies. For example, it highlights the need to intensify prevention efforts in rural areas and socioeconomically disadvantaged regions, with a particular focus on individuals with lower levels of education.

The identification of latent risk profiles in our study is consistent with findings from analyses using UK Biobank data.^25^ There, similar profiles—particularly those related to metabolic health and substance use—have been identified. The convergence of findings across datasets and contexts suggests that these risk constellations may reflect broader population‐level patterns. Future studies should explore the generalizability of these profiles across countries and cultural settings and test the feasibility and effectiveness of tailored intervention strategies for each subgroup.

### 4.1 Limitations

When calculating the prevention potential, several limitations must be considered: First, the estimation rests on the assumption that the included risk factors are causal drivers of dementia rather than prodromal markers of early disease. For several factors—such as depression, social isolation, and hearing loss—bidirectional associations and potential reverse causation are plausible, which could overestimate the preventable fraction. Second, we assume that the relative risks derived from the Lancet Commission's report^1^ are transferable to the German context. However, national differences in socioeconomic conditions, healthcare provision (e.g., hypertension control, hearing‐aid coverage), and diagnostic practices may modify effect sizes. Germany, for example, has a well‐developed education system with compulsory schooling. Even individuals classified as having “low education” may, on average, have more formal schooling than in countries with more limited access to education. Consequently, the relevant relative risks in Germany may differ and the resulting numbers of preventable cases should be interpreted with appropriate caution. Third, in its original form, Levin's formula determines the prevention potential in isolation for a single risk factor.^26^ In this analysis, we used a modified version with weighting factors to adjust for intercorrelations between risk factors.^16^ However, potential interactions between risk factors (including non‐modifiable ones) are not accounted for in the formula—this remains an area in need of further research.^27^ A fourth limitation concerns selection bias—although the aim of the DEAS is to achieve a representative sample of the population, certain groups tend to participate disproportionately often in surveys (e.g., individuals with higher educational attainment).^28^ In the DEAS as well, individuals with a low level of education are underrepresented. To correct for selection bias and still estimate the prevalence of dementia risk factors as accurately as possible, weights based on the German Microcensus were applied.

## 5 CONCLUSION

Our analyses indicate that there is a substantial potential for dementia prevention in Germany and that a considerable number of cases could be prevented or delayed through effective interventions. In addition, we identified distinct risk profiles within the population, each defined by specific constellations of modifiable risk factors and sociodemographic characteristics. These profiles offer a valuable foundation for developing more targeted and equitable prevention strategies that are tailored to the needs of different population subgroups. Future research should focus on testing the effectiveness and feasibility of targeted interventions for empirically identified subgroups, taking both risk exposure and access barriers into account.

## CONFLICT OF INTEREST STATEMENT

R.T. is a member of the boards of directors of the German Alzheimer Society (Deutsche Alzheimer Gesellschaft e. V.) and Alzheimer Europe. IB has no conflicts to disclose. Author disclosures are available in the Supporting Information.

## CONSENT STATEMENT

We used anonymized secondary data from the German Aging Survey (DEAS). All participants provided written informed consent at the time of data collection. The study procedures complied with applicable ethical standards and data protection regulations.

## Supporting information



Supporting information

Supporting information
