# Supplementary material for: Toward targeted dementia prevention: Population attributable fractions and risk profiles in Germany
Source: Alzheimers Dement (Amst). 2025 Nov 26;17(4):e70225. doi: 10.1002/dad2.70225 (PMC12657119; doi:10.1002/dad2.70225)
Supplement: Supplementary file 2 — Supporting information [file DAD2-17-e70225-s002.docx]

**Supplement**

**S1.** Overview of twelve modifiable risk factors for dementia and their definitions in DEAS 2023.

| Risk Factor | Definition |
| --- | --- |
| Less education | Highest educational attainment in category 0–2 according to the International Standard Classification of Education (ISCED) [1] from DEAS 2023 [2] |
| Hearing loss | 1) Self-reported hearing loss from DEAS 2023 [2]  2) Prevalence of hearing loss from GHS [3] |
| High LDL cholesterol | Self-reported high cholesterol in those under 65 years of age from DEAS 2023 [2] |
| Depression | 1) Lifetime prevalence from NAKO [4]: diagnostic criteria of major depression according to DSM-IV  2) Point prevalence from DEAS 2023 [2]: short version of the General Depression Scale (ADS-K), ≥ 18 points |
| Physical inactivity | Moderate or vigorous physical activity less than once a week from DEAS 2023 [2] |
| Smoking | Daily or occasional smoking from DEAS 2023 [2] |
| Diabetes | Self-reported diabetes mellitus from DEAS 2023 [2] |
| Hypertension | Self-reported hypertension in those under 65 years of age from DEAS 2023 [2] |
| Obesity | Body mass index of 30 or more in in those under 65 years of age from DEAS 2023 [2] |
| Alcohol consumption | Daily alcohol consumption or alcohol consumption several times per week from DEAS 2023 [2] |
| Social isolation | Regular contact with fewer than two significant others [5] from DEAS 2023 [2] |
| Visual impairment | Self-reported moderate to severe visual impairment [2] |

*Notes.* DEAS 2023 = German Aging Survey 2023, NAKO = NAKO Health Study, GHS = Gutenberg Health Study.

**S2.** Prevalences, relative risks, commonalities, population attributable fractions (PAF), and potential impact fractions (PIF, for a 15% or 30% prevalence reduction) for thirteen potentially modifiable risk factors (including crude estimates of air pollution)

| Risk factor | Prevalence in the population in Germany, % | Relative risk [95% CI] | Communality, % | PAF, % [95% CI] | Adjusted PAF, % [95% CI] | 15% reduction of risk factor prevalence | | 30% reduction of risk factor prevalence | |
| --- | --- | --- | --- | --- | --- | --- | --- | --- | --- |
|  |  |  |  |  |  | PIF, % [95% CI] | Adjusted PIF, % (95% CI) | PIF, % [95% CI] | Adjusted PIF, % (95% CI) |
| Less education | 15.4 | 1.6 (1.3-2.0) | 46.2 | 8.5 (4.4-13.3) | 3.3 (2.0-4.5) | 1.3 (0.7-2.0) | 0.6 (0.3-0.9) | 2.5 (1.3-4.0) | 1.1 (0.6-1.7) |
| Hearing loss | 23.3^1^ - 40.6^2^ | 1.4 (1.0-1.9) | 56.7 | 14.0 (0.0-26.8) | 5.4 (0.0-9.1) | 2.1 (0.0-4.0) | 0.9 (0.0-1.8) | 4.2 (0.0-8.0) | 1.8 (0.0-3.4) |
| High LDL cholesterol | 23.9 | 1.3 (1.3-1.4) | 43.5 | 6.7 (6.7-8.7) | 2.6 (3.0-3.0) | 1.0 (1.0-1.3) | 0.5 (0.5-0.6) | 2.0 (2.0-2.6) | 0.9 (1.0-1.1) |
| Depression | 10.3^3^ – 15.9^4^ | 2.2 (1.7-3.0) | 59.2 | 16.0 (10.0-24.1) | 6.2 (4.5-8.2) | 2.4 (1.5-3.6) | 1.1 (0.7-1.6) | 4.8 (3.0-7.2) | 2.1 (1.4-3.0) |
| Physical inactivity | 20.7 | 1.2 (1.2-1.3) | 33.3 | 4.0 (4.0-5.8) | 1.5 (1.8-2.0) | 0.6 (0.6-0.9) | 0.3 (0.3-0.4) | 1.2 (1.2-1.8) | 0.5 (0.6-1.0) |
| Smoking | 22.3 | 1.3 (1.2-1.4) | 55.3 | 6.3 (4.3-8.2) | 2.4 (1.9-2.8) | 0.9 (0.6-1.2) | 0.4 (0.3-0.5) | 1.9 (1.3-2.5) | 0.8 (0.6-1.0) |
| Diabetes | 11.1 | 1.7 (1.6-1.8) | 51.3 | 7.2 (6.2-8.2) | 2.8 (2.8-2.8) | 1.1 (0.9-1.2) | 0.5 (0.5-0.5) | 2.2 (1.9-2.4) | 1.0 (0.9-1.0) |
| Hypertension | 34.5 | 1.2 (1.1-1.4) | 60.0 | 6.5 (3.3-12.1) | 2.5 (1.5-4.1) | 1.0 (0.5-1.8) | 0.4 (0.2-0.8) | 1.9 (1.0-3.6) | 0.9 (0.5-1.5) |
| Obesity | 26.7 | 1.3 (1.0-1.7) | 59.5 | 7.4 (0.0-15.7) | 2.9 (0.0-5.4) | 1.1 (0.0-2.4) | 0.5 (0.0-1.0) | 2.2 (0.0-4.7) | 1.0 (0.0-2.0) |
| Alcohol consumption | 29.2 | 1.2 (1.0-1.5) | 60.7 | 5.5 (0.0-12.7) | 2.1 (0.0-4.3) | 0.8 (0.0-1.9) | 0.4 (0.0-0.8) | 1.7 (0.0-3.8) | 0.7 (0.0-1.6) |
| Social isolation | 8.9 | 1.6 (1.3-1.8) | 31.8 | 5.1 (2.6-6.6) | 2.0 (1.2-2.3) | 0.8 (0.4-1.0) | 0.3 (0.2-0.4) | 1.5 (0.8-2.0) | 0.7 (0.4-0.8) |
| Air pollution | 60.3 | 1.1 (1.1-1.1) | 9.1 | 5.7 (4.6-6.8) | 2.4 (2.4-2.4) | 0.9 (0.9-0.9) | 0.4 (0.4-0.4) | 1.7 (1.7-1.7) | 0.8 (0.8-0.8) |
| Visual impairment | 12.1 | 1.5 (1.4-1.6) | 62.0 | 5.7 (4.6-6.8) | 2.2 (2.1-2.3) | 0.9 (0.7-1.0) | 0.4 (0.3-0.4) | 1.7 (1.4-2.0) | 0.8 (0.7-0.8) |
| Total |  |  |  |  | 39.0  (24.7-53.3) |  | 7.0  (4.1-10.5) |  | 13.6  (8.1-20.0) |

Notes: Commonality represents the proportion of shared variance of the risk factor in question and the other risk factors. Percentages are based on exact values, minor deviations from the total are due to rounding. ^1^ Prevalence in the German Aging Survey [2], ^2^ Prevalence based on pure-tone audiometric assessments from the Gutenberg Health Study [3], ^3^ Point prevalence from the German Aging Survey [2], ^4^ Lifetime prevalence from the NAKO Health Study [4]; CI, confidence interval

**S3.** Goodness-of-fit measures and estimated class-specific probabilities of the five investigated models for deciding the number of classes

| Number of classes | Goodness-of-fit measures | | | | | | Average latent class probabilities for most likely class membership | | | | |
| --- | --- | --- | --- | --- | --- | --- | --- | --- | --- | --- | --- |
|  | Entropy | AIC | BIC | ssABIC | VLMR | LMRT | Class 1 | Class 2 | Class 3 | Class 4 | Class 5 |
| 1 | - | 138372 | 138561 | 138469 | - | - | 1.000 | - | - | - | - |
| 2 | 0.503 | 46842 | 47050 | 46948 | *p* < 0.001 | *p* < 0.001 | 0.851 | 0.837 | - | - | - |
| 3 | 0.573 | 46283 | 46629 | 46460 | *p* < 0.001 | *p* < 0.001 | 0.789 | 0.838 | 0.774 | - | - |
| 4 | 0.553 | 45988 | 46470 | 46235 | *p* = 0.111 | *p* = 0.112 | 0.741 | 0.752 | 0.682 | 0.800 | - |
| 5 | 0.550 | 45846 | 46465 | 46163 | *p* = 0.450 | *p* = 0.452 | 0.715 | 0.733 | 0.692 | 0.742 | 0.670 |

*Notes.* ssABIC = sample-size adjusted BIC, VLMR = Vuong-Lo-Mendell-Rubin likelihood ratio test, LMRT = Lo-Mendell-Rubin adjusted likelihood ration test

**S4.** Sample characteristics and characterization of risk factor patterns

|  | Total  (n = 4989) | Class 1 „Metabolic syndrome“  (n = 876, 17.6%) | Class 2 „Sensory impairment“  (n = 1122, 22.5%) | Class 3 „Alcohol consumption“  (n = 1181, 23.7%) | Class 4 „Lower risk“  (n = 1810, 36.3%) | *p* |
| --- | --- | --- | --- | --- | --- | --- |
| Age, M (SD) | 69.0 (11.6) | 67.4 (8.8) | 82.5 (5.5) | 70.7 (7.3) | 60.3 (9.5) | < 0.001 |
| Female Sex, n (%) | 2600 (52.1) | 527 (60.2) | 598 (53.3) | 212 (18.0) | 1263 (69.8) | < 0.001^b^ |
| Education level |  |  |  |  |  | < 0.001^b^ |
| Low education, % (ref) | 197 (3.9) | 71 (8.1) | 57 (5.1) | 19 (1.6) | 50 (2.8) |  |
| Moderate education, n (%) | 2334 (46.8%) | 572 (65.3%) | 555 (49.5%) | 312 (26.4%) | 895 (49.4%) |  |
| High education, n (%) | 2458 (49.3%) | 233 (26.6%) | 510 (45.5%) | 850 (72%) | 865 (47.8%) |  |
| Cohabitating, n (%) | 3186 (63.9%) | 521 (59.5%) | 589 (52.5%) | 1061 (89.8%) | 1015 (56.1%) | < 0.001^b^ |
| Eastern Germany, n (%) | 1539 (30.8%) | 379 (43.3%) | 409 (36.5%) | 269 (22.8%) | 482 (26.6%) | < 0.001^b^ |
| District type |  |  |  |  |  |  |
| Independent large cities | 1385 (27.8) | 186 (21.2) | 323 (28.8) | 317 (26.8) | 559 (30.9) | < 0.001^b^ |
| Urbanized counties | 1700 (34.1) | 252 (28.8%) | 369 (32.9%) | 492 (41.7%) | 587 (32.4%) |  |
| Semi urban-rural districts | 958 (19.2) | 174 (19.9%) | 225 (20.1%) | 201 (17%) | 358 (19.8%) |  |
| Sparsely populated rural districts | 946 (19.0) | 264 (30.1%) | 205 (18.3%) | 171 (14.5%) | 306 (16.9%) |  |
| Risk factors |  |  |  |  |  |  |
| Hearing loss | 1209 (24.2%) | 174 (19.9%) | 573 (51.1%) | 322 (27.3%) | 140 (7.7%) | < 0.001^b^ |
| High LDL cholesterol | 1680 (33.7%) | 461 (52.6%) | 441 (39.3%) | 507 (42.9%) | 271 (15%) | < 0.001^b^ |
| Depression | 334 (6.7%) | 136 (15.5%) | 83 (7.4%) | 9 (0.8%) | 106 (5.9%) | < 0.001^b^ |
| Physical inactivity | 726 (14.6%) | 181 (20.7%) | 288 (25.7%) | 73 (6.2%) | 184 (10.2%) | < 0.001^b^ |
| Smoking | 583 (11.7%) | 137 (15.6%) | 11 (1%) | 127 (10.8%) | 308 (17%) | < 0.001^b^ |
| Diabetes | 663 (13.3%) | 335 (38.2%) | 173 (15.4%) | 131 (11.1%) | 24 (1.3%) | < 0.001^b^ |
| Hypertension | 2443 (49%) | 796 (90.9%) | 682 (60.8%) | 723 (61.2%) | 242 (13.4%) | < 0.001^b^ |
| Obesity | 979 (19.6%) | 591 (67.5%) | 61 (5.4%) | 163 (13.8%) | 164 (9.1%) | < 0.001^b^ |
| Alcohol consumption | 1494 (29.9%) | 73 (8.3%) | 218 (19.4%) | 824 (69.8%) | 379 (20.9%) | < 0.001^b^ |
| Social isolation | 458 (9.2%) | 89 (10.2%) | 155 (13.8%) | 88 (7.5%) | 126 (7%) | < 0.001^b^ |
| Visual impairment | 491 (9.8%) | 86 (9.8%) | 271 (24.2%) | 45 (3.8%) | 89 (4.9%) | < 0.001^b^ |

*Notes.* Latent Class Analysis was based on N = 4989 due to three missing values in the covariate education. ^a^ Analysis of Variance, ^b^ Chi²-Test; M, Mean; SD, Standard Deviation.

**References**

1. UNESCO. International Standard Classification of Education, ISCED 1997 Springer US; 2003 (pp. 195-220).

2. Deutsches Zentrum für Altersfragen (DZA). Deutscher Alterssurvey (SUF DEAS 2023), doi: 10.5156/DEAS.2023.D.001. 2025.

3. Döge J, Hackenberg B, Bohnert A, Bahr K, Matthias C. Prävalenz von Schwerhörigkeiten sowie deren Hörgeräteversorgung bzw.-Unterversorgung im Großraum Mainz. Laryngo-Rhino-Otologie. 2022;101(S 02).

4. Streit F. Lifetime and current depression in the German National Cohort (NAKO). World J Biol Psychiatry. 2022:1-16.

5. Huxhold O, Engstler H. Soziale Isolation und Einsamkeit bei Frauen und Männern im Verlauf der zweiten Lebenshälfte. Frauen und Männer in der zweiten Lebenshälfte: Springer; 2019. p. 71-89.
